# Everolimus in metastatic renal cell carcinoma after failure of initial anti–VEGF therapy: final results of a noninterventional study

**DOI:** 10.1186/s12885-015-1309-7

**Published:** 2015-04-18

**Authors:** Lothar Bergmann, Ulrich Kube, Christian Doehn, Thomas Steiner, Peter J Goebell, Manfred Kindler, Edwin Herrmann, Jan Janssen, Steffen Weikert, Michael T Scheffler, Joerg Schmitz, Michael Albrecht, Michael Staehler

**Affiliations:** 1 Medical Clinic II, J. W., Goethe University Frankfurt, Theodor-Stern-Kai 7, Frankfurt/Main, 60590 Germany; 2 Private Practice for Urology, Goethestrasse 5, 09119 Chemnitz, Germany; 3 Urologikum Luebeck, Am Kaufhof 2, 23566 Luebeck, Germany; 4 Department of Urology, Hospital Erfurt, Nordhaeusertrasse 74, 99089 Erfurt, Germany; 5 Department of Urology, University Hospital Erlangen, Rathsberger Strasse 57, 91054 Erlangen, Germany; 6 Private Practice for Oncology, Landsberger Allee 277a, 13055 Berlin, Germany; 7 Department of Urology, University Hospital Muenster, Albert Schweitzer Campus 1, Gebaeude A1, 48149 Muenster, Germany; 8 Private Practice for Oncology, Kuhlenstrasse 53d, 26655 Westerstede, Germany; 9 Department of Urology, Vivantes Humboldt-Hospital and Charité-University Medicine, Am Nordgraben 2, 13509 Berlin, Germany; 10 Private Practice for Urology, Friedrich Staude Strasse 2, 08060 Zwickau, Germany; 11 St. Johannes-Hospital, Springufer 7, 59755 Arnsberg, Germany; 12 Novartis Pharma GmbH, Roonstrasse 25, 90429 Nuremberg, Germany; 13 Department of Urology, University Hospital Munich-Grosshadern, Marchioninistrasse, 15, 81377 Germany

**Keywords:** Observational study, Everolimus (RAD001), Carcinoma, Renal cell, Targeted molecular therapy

## Abstract

**Background:**

Data are limited regarding routine use of everolimus after initial vascular endothelial growth factor (VEGF)–targeted therapy. The aim of this prospective, noninterventional, observational study was to assess efficacy and safety of everolimus after initial VEGF-targeted treatment in patients with metastatic renal cell carcinoma (mRCC) in routine clinical settings.

**Methods:**

Everolimus was administered per routine clinical practice. Patients with mRCC of any histology from 116 active sites in Germany were included. The main objective was to determine everolimus efficacy in time to progression (TTP). Progression-free survival (PFS), treatment duration, tumor response, adherence to everolimus regimen, treatment after everolimus, and safety were also assessed.

**Results:**

In the total population (N = 334), median follow-up was 5.2 months (range, 0–32 months). Median treatment duration (safety population, n = 318) was 6.5 months (95% confidence interval [CI], 5–8 months). Median TTP and median PFS were similar in populations investigated. In patients who received everolimus as second-line treatment (n = 211), median (95% CI) TTP was 7.1 months (5–9 months) and median PFS was 6.9 months (5–9 months). Commonly reported adverse events (safety population, n = 318) were dyspnea (17%), anemia (15%), and fatigue (12%). Limitations of the noninterventional design should be considered.

**Conclusions:**

This study reflects routine clinical use of everolimus in a large sample of patients with mRCC. Favorable efficacy and safety were seen for everolimus after previous therapy with one VEGF-targeted agent. Results of this study confirm everolimus as one of the standard options in second-line therapy for patients with mRCC. Novartis study code, CRAD001LD27: VFA registry for noninterventional studies (http://www.vfa.de/de/forschung/nisdb/).

**Electronic supplementary material:**

The online version of this article (doi:10.1186/s12885-015-1309-7) contains supplementary material, which is available to authorized users.

## Background

In the European Union, the mammalian target of rapamycin (mTOR) inhibitor everolimus (Afinitor; Novartis, Basel, Switzerland) is registered for treatment of patients with metastatic renal cell carcinoma (mRCC) after failure of a previous vascular endothelial growth factor (VEGF)–targeted agent (VEGF antibody or VEGF receptor-tyrosine kinase inhibitor [VEGFR-TKI]) [1]. Approval was based on results of the phase 3 RECORD-1 study in which everolimus significantly improved median progression-free survival (PFS) compared with placebo (4.9 vs. 1.9 months; hazard ratio [HR], 0.33; *p* < .001) in patients who previously received sunitinib, sorafenib, or both (previous cytokines and/or bevacizumab also permitted); 21% of patients previously received one medication before everolimus [2]. A subgroup analysis of RECORD-1 showed numerically longer median PFS in patients who previously received only one VEGFR-TKI than in patients who previously received two VEGFR-TKIs (5.4 and 4.0 months, respectively) [3]. Median PFS of patients who previously received sunitinib as the only antineoplastic treatment was 4.6 months with everolimus (n = 43) and 1.8 months with placebo (n = 13) (hazard ratio [HR], 0.22; *p* < .001) [3]. RECORD-1 showed a favorable tolerability profile for everolimus, with a low rate of grade 3 or 4 adverse events (AEs) and low rates of dose modification (7%) and treatment discontinuation because of AEs (13%) [2]. Results of the international, open-label, expanded-access program REACT were consistent with results of RECORD-1 and showed that everolimus was well tolerated and provided clinical benefit (52% stable disease) in VEGFR-TKI–refractory patients with mRCC [4].

A recently published retrospective analysis investigated the efficacy of sequential VEGFR-TKI, VEGFR-TKI, and mTOR inhibitor and of sequential VEGFR-TKI, mTOR inhibitor, and VEGFR-TKI in Italy [5]. Median PFS ranged from 36.5 to 29.3 months, and median overall survival (OS) ranged from 50.7 to 37.8 months. The study was performed in a nonrandomized, retrospective setting based on a highly selected patient population (only 13% of all treated patients had received three lines of targeted therapy). Because of potential immortal time bias for results of second-line treatment, this study did not meet the requirements for inclusion in a meta-analysis of adjusted, multicenter, retrospective cohort studies, which showed that OS was significantly prolonged in VEGFR-TKI–refractory patients with mRCC treated with a second-line mTOR inhibitor compared with a second-line VEGFR-TKI (HR, 0.82; 95% confidence interval [CI], 0.68–0.98) [6]. Although these studies and analyses added insight into sequential treatment options for patients with mRCC, data regarding the routine use of everolimus in second-line therapy after initial VEGF-targeted therapy still are limited. Therefore, this noninterventional study assessed the efficacy and safety of everolimus after initial VEGF-targeted therapy in patients with mRCC in the routine clinical setting in Germany.

## Methods

### Study design

This was a prospective, observational study conducted at 166 registered sites. Patients with mRCC (clear cell or non–clear cell) were enrolled when the physician intended to treat them with everolimus after failure of one VEGF-targeted therapy (VEGFR-TKI or bevacizumab). To ensure mainly prospective observation, retrospective enrollment was limited to patients who had begun treatment with everolimus up to 90 days before the start of the study or had undergone no more than one image-based follow-up since treatment initiation. Everolimus was administered according to the approved product label in Europe [7]. Patients received everolimus 10 mg once daily until disease progression or unacceptable toxicity. Dose interruptions, reductions to 5 mg/day, or both were used, if necessary, to manage side effects.

The study was performed in accordance with German drug law and relevant guidelines of the German health authorities and the pharmaceutical industry for conducting noninterventional studies. The ethics committee of the Johann Wolfgang Goethe-University Frankfurt am Main, which was constituted according to state law and bears responsibility for the medical leader of this study, granted approval of the observational plan. Patients provided written informed consent before the start of the study.

### Aim and objectives

The aim of the study was to estimate the efficacy and safety of everolimus after the first anti-VEGF agent in routine clinical practice. The main objective was to determine everolimus efficacy in terms of time to progression (TTP; time between baseline and progression based on physician assessment). In addition, PFS (time between baseline and progression or death based on physician assessment or death from any cause) was assessed. Patients who did not experience progression and who did not die during the observation period were censored at study discontinuation; patients without a documented study discontinuation date were censored at the analysis cutoff date. Other objectives included treatment duration, tumor response, adherence to everolimus, posteverolimus treatment, and safety.

### Assessments

Evaluation of treatment response and progression was based on physician assessment (i.e., on clinical judgment and/or imaging results; Response Evaluation Criteria In Solid Tumors [RECIST] evaluation was possible but not mandatory). In accordance with routine practice, documentation of the following observation parameters was aimed for regular visits, in line with routine practice (e.g., after approximately 3-month intervals): assessment of the response to treatment (computed tomography/magnetic resonance imaging (CT/MRI), skeletal scintigraphy, positron emission tomography (PET)/PET-CT, ultrasound) and/or clinical assessment of the patient’s status. Kaplan-Meier statistics were applied for analysis of treatment duration, TTP, PFS, and time to worsening of Karnofsky performance status (KPS); in cases of descriptive comparisons of such parameters, the log-rank test was used. For all other parameters, descriptive statistics were applied. AEs were collected and coded to a preferred term using the Medical Dictionary for Regulatory Activities (MeDRA). According to the methodological features of an observational noninterventional study, all statistical analyses were descriptive, and the presented results should be interpreted as such.

### Patients

**Figure 1:**
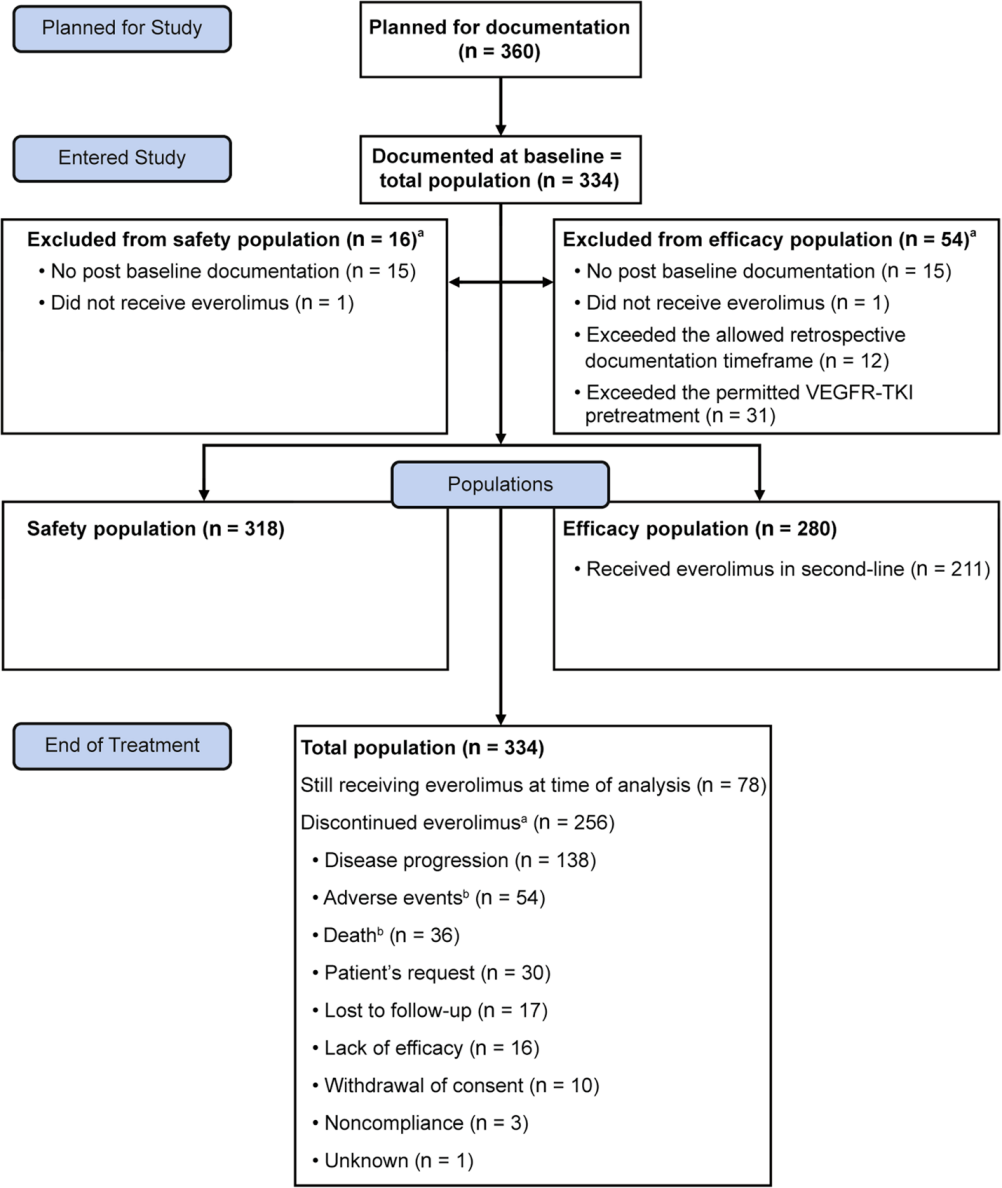
CHANGE patient disposition. VEGFR-TKI, vascular endothelial growth factor receptor–tyrosine kinase inhibitor. ^a^More than one reason per patient. ^b^Adverse event and death were reasons for discontinuation for four patients.

The study was planned to enroll 360 patients; at the end of observation, 334 patients had been enrolled (Figure 1). The first interim analysis was based on all patients with ≥3 months of documented evaluation or discontinuation and included 113 patients (median observation, 3.9 months) [8]. The second interim analysis was performed after patients from the first analysis were followed up for another 10 months and included those patients plus patients who had entered the trial at this stage (N = 196; median observation, 4.7 months) [9].

The total population included all patients who were enrolled at baseline. The safety population included all patients from the total population who had documented evidence of everolimus intake and one or more postbaseline assessments. The efficacy population included all patients from the safety population who were enrolled ≤90 days after the initiation of treatment and who received a single VEGF-targeted therapy (VEGFR-TKI or bevacizumab); a second VEGFR-TKI was allowed for <1 month before everolimus. The population receiving everolimus after receiving exactly one previous VEGF-targeted therapy (i.e., everolimus in second line) included patients from the efficacy population who previously received exactly one VEGF-targeted agent (Figure 1).

## Results

### Patients and treatment duration

The observation period was from August 17, 2009, to January 18, 2013. The total population was composed of 334 patients enrolled at 116 active sites. The safety population included 318 patients, the efficacy population included 280, and the population receiving everolimus as second-line treatment included 211 (Figure 1). Among patients in the total population (multiple items possible per patient), there was no further documented evaluation after baseline for 15 patients, 1 did not receive everolimus, 12 exceeded the allowed retrospective enrollment time frame, and 31 exceeded the permitted VEGFR-TKI pretreatment. In the total population, median follow-up was 5.2 months (range, 0–32 months); median follow-up time under treatment was 22.4 months (range, 12–41 months) for 78 patients still receiving treatment at the end of observation.

Median patient age was 68 years (range, 22–89 years), and median KPS at baseline was 80% (range, 50–100%) (Table 1). Of the patients, 75% were men, 88% had clear cell histology, and 92% had favorable/intermediate Memorial Sloan-Kettering Cancer Center (MSKCC) risk (at first-line therapy). Most patients (72%) previously received only one systemic therapy. The most common previous targeted agent was sunitinib (78%), for which median treatment duration was 9 months (range, 0–63 months) (Table 2). In the overall population, 86% of patients switched from their previous therapy to everolimus because of progression, and 14% switched because of other reasons (e.g., intolerance or patient request).

**Table 1:** **Baseline characteristics**

Characteristics	Total population (N = 334)
Age, median (range), y	68 (22–89)
Sex, n (%)	
Men	250 (75)
Women	84 (25)
Tumor histology, n (%)	
Clear cell	293 (88)
Non–clear cell	24 (7)
Missing	17 (5)
KPS, median (range)	80 (50–100)
Time since diagnosis, median (range), y	
Initial	3.3 (0–34)
Metastasis	1.7 (0–16)
MSKCC risk status at start of first-line therapy, n (%)^a^	
Favorable	84 (35)
Intermediate	134 (56)
Poor	20 (8)
Primary metastatic site, n (%)^b^	
Lung	226 (68)
Lymph node	145 (43)
Skeletal system	125 (37)
Liver	87 (26)
Adrenal gland	47 (14)
Previous surgery, n (%)	325 (97)
Previous nephrectomy	300 (90)
Number of previous antineoplastic therapies, n (%)	
1	240 (72)
2	69 (21)
≥3	25 (7)

Abbreviations: MSKCC, Memorial Sloan-Kettering Cancer Center; KPS, Karnofsky performance status.

^a^100% relate to patients with documented evaluation (n = 238).

^b^Patients could have had multiple metastatic locations.

**Table 2:** **Previous therapy, targeted agents, and cytokines (total population, N = 334)**

Previous therapy^a^	Patients, n (%)	Duration of treatment,^b^median (range), mo
Sunitinib	260 (78)	9 (0–63)
Sorafenib	68 (20)	6 (0–48)
Pazopanib	12 (4)	3 (1–11)
Bevacizumab^c^	41 (12)	6 (0–29)
Cytokines^d^	33 (10)	8 (0–113)

^a^Patients could have received multiple previous therapies.

^b^Duration was calculated for patients with information on duration (sunitinib, n = 251; sorafenib, n = 65; pazopanib, n = 12; bevacizumab, n = 41; cytokines, n = 33).

^c^Given as monotherapy in 14 patients and as part of combination therapy in 27 patients.

^d^Combination of cytokines and bevacizumab was included in the bevacizumab category.

Among patients who were evaluated at visits 2–6 and for whom data were accessible, imaging was used in at least 46% and at most 73% of patients per visit in the safety population and in at least 40% and at most 72% of patients per visit in the efficacy population. Likewise, during visits 2–6, RECIST criteria were applied to at least 46% and at most 63% of patients per visit in the safety population and in at least 25% and at most 59% of patients per visit in the efficacy population.

In the safety population, 138 patients discontinued because of progression and, of those patients, progression was documented by imaging in 81%. Similarly, among 115 patients in the efficacy population who discontinued because of progression, imaging documented progression in 78%.

**Figure 2:**
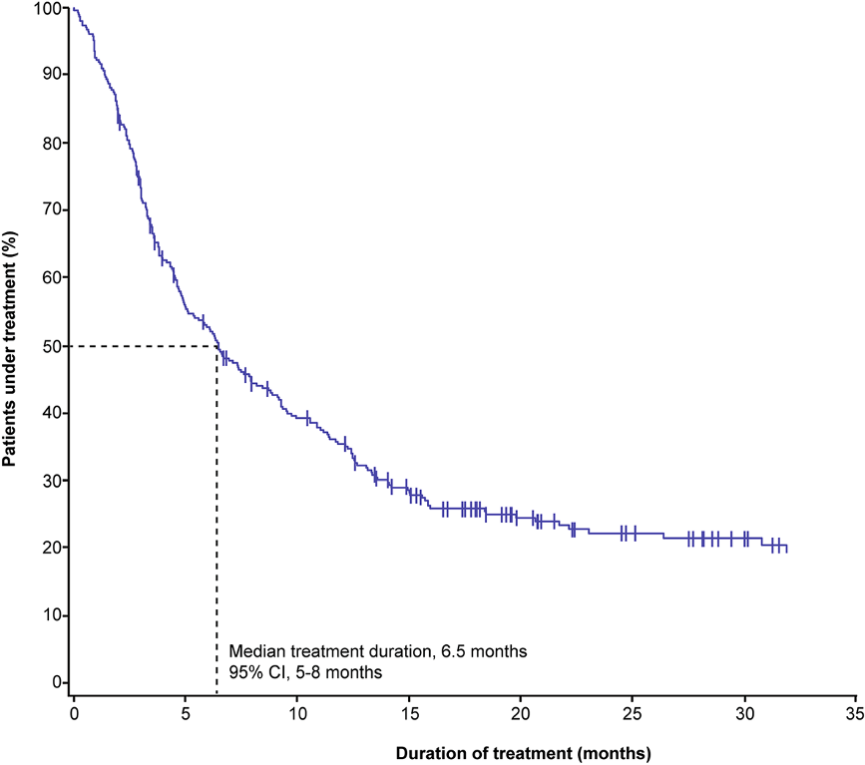
Duration of everolimus treatment in the safety population (n = 318). CI, confidence interval.

Median duration of everolimus treatment was 6.5 months (95% CI, 5–8 months; mean dose intensity, 94%) in the safety population (Figure 2), 6.6 months (95% CI, 5–8 months) in the efficacy population, and 6.6 months (95% CI, 5–9 months) in the population receiving everolimus as second-line treatment.

### Efficacy

**Figure 3:**
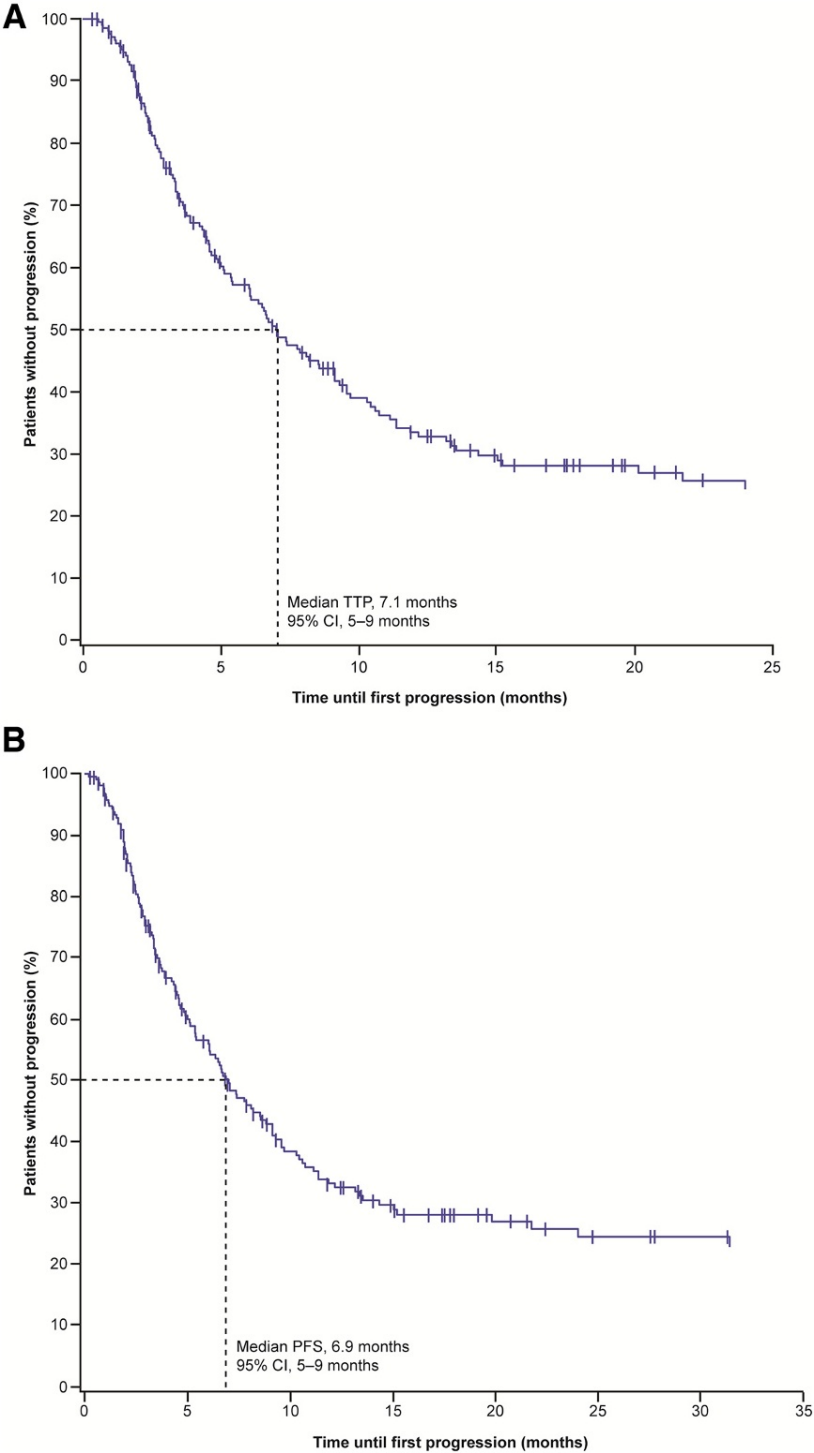
TTP **(A)** and PFS **(B)** (population of patients receiving everolimus as second-line treatment, n = 211). PFS, progression-free survival; TTP, time to progression.

Median TTP was 7.0 months (95% CI, 6–9 months) in the safety population, 7.4 months (95% CI, 6–9 months) in the efficacy population, and 7.1 months (95% CI, 5–9 months) in the population of patients who received everolimus as second-line treatment (Figure 3A). Similar to TTP, median PFS was 6.9 months (95% CI, 5–9 months) in the population of patients who received everolimus as second-line treatment; (Figure 3B). Best overall response (efficacy population, 217 patients with assessable data) was remission in 13.8% of patients, stable disease in 57.1%, and disease progression in 29.0%.

### Safety

**Figure 4:**
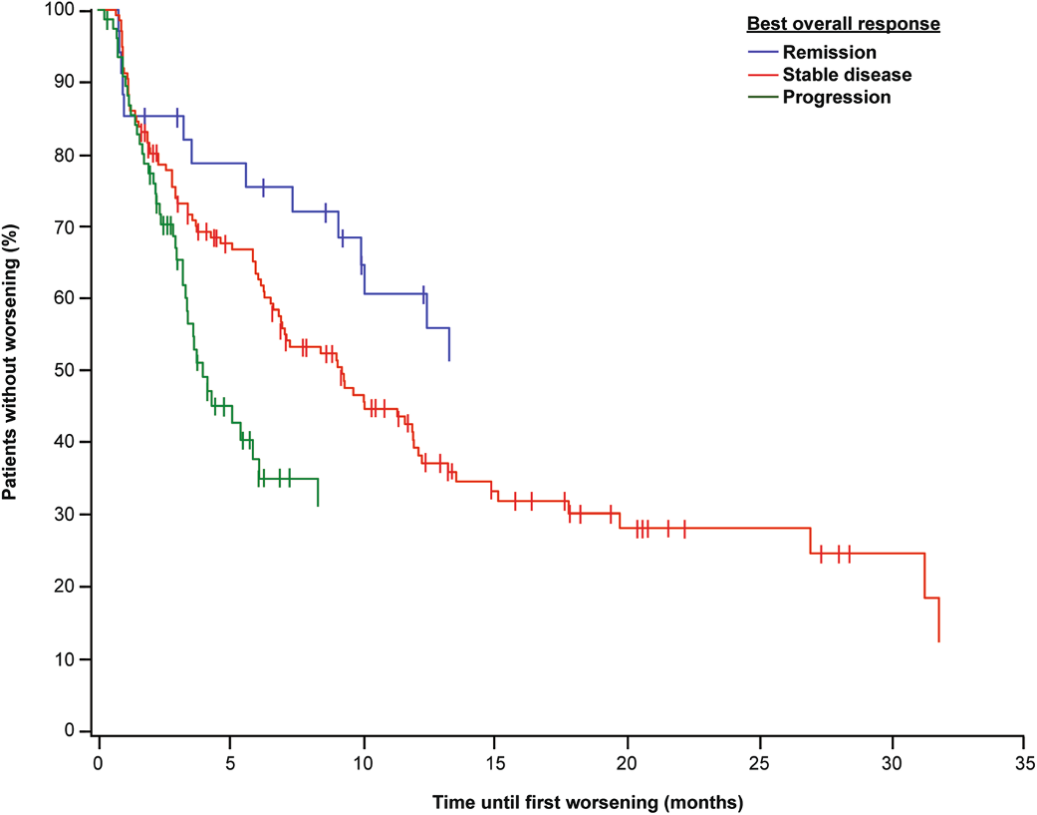
Time to worsening of Karnofsky performance status by ≥10% by best overall response (safety population). Remission, n = 29; stable disease, n = 124; disease progression, n = 68.

Adverse events that occurred in ≥5% of patients in the safety population and the corresponding rate of serious AEs are shown in Table 3. The most commonly reported AEs were dyspnea (17%), anemia (15%), and fatigue (12%). Pneumonitis occurred in 4% of patients (n = 14). Twenty-seven percent (n = 85) of patients required dose adjustment, and 20% (n = 63) required dose interruption (median duration, 14 days [range, 2–90 days]). Median time to worsening of KPS by ≥10% from baseline was 8.4 months (95% CI, 6–10 months) overall, not reached (NR; 95% CI, 9 months–NR) for patients with remission, 9.3 months (95% CI, 7–12 months) for patients with stable disease, and 4 months (95% CI, 3–6 months) for patients with progression (Figure 4). Among patients in the safety population, 18% (n = 57) died. AEs potentially related to study treatment were observed in 11 patients who died. However, in nine patients, a tumor-related cause of death was documented; one patient died of acute myocardial infarction; and one died of acute renal failure.

**Table 3:** **Adverse events irrespective of suspected causality with everolimus that occurred in ≥5% of patients (safety population, n = 318)**

Adverse event^a^	All events, n (%)	Serious events, n (%)
Overall	224 (70)	125 (39)
Dyspnea	54 (17)	31 (10)
Anemia	46 (15)	21 (7)
Fatigue	37 (12)	13 (4)
Cough	33 (10)	19 (6)
Nausea	28 (9)	6 (2)
Pain	24 (8)	6 (2)
General physical health deterioration	23 (7)	19 (6)
Stomatitis	22 (7)	4 (1)
Peripheral edema	21 (7)	10 (3)
Mucositis	19 (6)	1 (<1)
Pyrexia	19 (6)	11 (4)
Rash	18 (6)	4 (1)
Decreased appetite	17 (5)	1 (<1)
Diarrhea	16 (5)	5 (2)
Decreased weight	16 (5)	4 (1)

^a^Disease progression was collected as an adverse event according to the requirement of observational plan. Progression-related events (neoplasm progression, malignant neoplasm, malignant neoplasm progression) were summarized under the preferred term “neoplasm progression”. Neoplasm progression was reported as an adverse event for 57 patients (18%) and as a serious event for 54 patients (17%).

### End of treatment

At the time of analysis, 78 patients (23%) of the total population were receiving everolimus and 256 patients (77%) had discontinued study treatment. The most common reasons for treatment discontinuation were disease progression (54%), AEs (21%), and death (14%) (See supplementary materials for reasons for disconnection of study treatment [Additional file 1: Table S1]). Among patients who discontinued treatment, 123 (48%) received subsequent therapy, most commonly sorafenib (36%), sunitinib (24%), or pazopanib (21%). Of patients who died (n = 57, safety population), AEs potentially related to study treatment were observed in 11 patients; however, in nine of these cases, a tumor-related cause of death was documented. Reported causes of death for two patients were acute myocardial infarction and acute renal failure.

### Subgroup analyses

Median TTP of everolimus treatment in second-line treatment after sunitinib (efficacy population, n = 188) was 7.1 months (95% CI, 5–9 months). Longer duration of previous VEGF-targeted therapy (VEGFR-TKI or bevacizumab) tended to result in longer median TTP with second-line everolimus; however, no statistically significant differences were observed (Table 4). (See supplementary materials for treatment duration, TTP, and PFS by MSKCC risk [Additional file 1: Table S2] and histology [Additional file 1: Table S3].)

**Table 4:** **Median treatment duration, median TTP, and median PFS of everolimus by duration of previous VEGF-targeted treatment (efficacy population)**

Previous VEGF-targeted treatment	Treatment duration	TTP	PFS
Duration, months	Median, months (95% CI)		
<3 (n = 54) vs. ≥3 (n = 203)	6.6 (3–10)	7.9 (4–11)	7.1 (4–11)
	7.1 (5–9)	7.5 (6–10)	7.4 (6–9)
		*p* = .87	*p* = .62
<6 (n = 105) vs. ≥6 (n = 152)	6.6 (4–9)	6.8 (4–10)	6.6 (4–10)
	7.5 (5–11)	8.1 (7–10)	7.8 (5–10)
		*p* = .86	*p* = .70
<9 (n = 133) vs. ≥9 (n = 124)	6.6 (4–9)	6.8 (4–10)	6.6 (4–10)
	7.5 (5–11)	8.2 (7–11)	8.1 (7–10)
		*p* = .79	*p* = .74

Abbreviations: CI, confidence interval; PFS, progression-free survival; TTP, time to progression; VEGF, vascular endothelial growth factor.

*p* value determined using log-rank test.

## Discussion

Current guidelines recommend second-line everolimus and axitinib after previous VEGFR-TKI therapy as a standard treatment option for patients with clear cell mRCC [10-12]. Although sequential therapy is the current standard of care, the optimal sequence has not been established. For example, results of the AXIS and INTORSECT trials showed clinical benefit of a second-line VEGFR-TKI in patients for whom first-line sunitinib was ineffective. In AXIS, second-line median PFS was 4.8 months for axitinib and 3.4 months for sorafenib in patients previously treated with sunitinib [13]; the corresponding median OS was 15.2 months for axitinib and 16.5 months for sorafenib [14]. In INTORSECT, second-line median PFS and OS were 3.9 and 16.6 months, respectively, in the sorafenib arm [15]. In addition, results of the SWITCH trial showed comparable median PFS (including first-line and second-line treatment; range, 12.5–14.9 months) and median OS (range, 30.2–31.5 months) for first-line sunitinib followed by sorafenib and first-line sorafenib followed by sunitinib [16]. Conversely, results of the large phase 2 trial RECORD-3, which investigated sunitinib followed by everolimus compared with the opposite sequence, support the treatment sequence of first-line sunitinib followed by second-line everolimus with a median combined PFS (including first-line and second-line treatment) of 25.8 months and a median OS of 32.0 months [17]. Taken together, results of these clinical trials indicate that the optimal sequence of targeted therapy must still be determined. Results of these clinical trials influence the interpretation of results of noninterventional studies, which are important for assessing sequentially administered targeted therapy in the daily routine setting. In addition, evidence of the clinical benefit of sequential VEGFR-TKI, mTOR inhibitor, and VEGFR-TKI therapy is increasing [18,19].

In the current study, median PFS was 6.9 months for patients who previously received exactly one VEGF-targeted agent and was 7.0 months for patients who previously received sunitinib only. In RECORD-1, median PFS was 4.9 months for the overall population [2] and 5.4 months for patients who previously received one VEGFR-TKI [3]. Although CHANGE was a noninterventional study with limitations inherent to its noninterventional character (assessment times according to daily practice; clinical response assessment allowed, RECIST evaluation not mandatory), treatment duration, deterioration of KPS, response, and TTP/PFS showed a high level of consistency, reflecting clinically relevant outcomes and clinical reality.

Longer duration of first-line therapy seems to correspond with improved effectiveness of second-line targeted agents. In the current study, second-line everolimus treatment resulted in favorable efficacy, with a median TTP of 6.8–8.2 months in patients with shorter or longer pretreatment duration. However, the longest median TTP (8.2 months) was observed in patients in whom pretreatment duration was longest (≥9 months). The current study results are in line with those of an AXIS subgroup analysis, which showed a trend toward longer PFS for patients who received axitinib after ≥9 months of sunitinib [20]. Moreover, longer first-line treatment with sunitinib or cytokines resulted in longer OS during second-line treatment with axitinib or sorafenib [20]. The reason for this potential correlation between previous treatment duration and second-line efficacy is not known. However, it seems plausible that longer first-line treatment duration corresponds to less aggressive tumors that respond better to second-line therapy. Although the current study results suggest a correlation between longer previous treatment duration and everolimus efficacy, they also show that everolimus is effective regardless of first-line therapy duration.

Results of RECORD-1 and REACT showed that everolimus is well tolerated in patients with mRCC after ineffective VEGF-targeted therapy [2,4]. The safety profile of everolimus observed in the current study in routine conditions was manageable and consistent with that of previous reports.

Limitations of the noninterventional design should be considered. Data validation by site monitoring visits occurred in 32% of the total population. We assume this rate is higher than the rate in many other comparable noninterventional studies, leading to higher documentation quality. However, the rate is lower than the rate in interventional trials, leading to lower data quality in comparison with this type of design. In addition, because of the noninterventional design (observation of routine procedures), assessment and assessment times cannot be standardized for study purposes. This is different than in interventional trials, in which standardized assessments are performed at predefined intervals. However, results of the current study are more representative of the effectiveness of everolimus in daily practice than are results from interventional trials because there was no comparable patient selection in interventional designs.

## Conclusions

This noninterventional study reflects the routine clinical use of everolimus in a large sample of patients with mRCC. Favorable efficacy and safety were shown for patients treated with everolimus after previous therapy with one VEGF-targeted agent. Results of this study confirm everolimus as one of the standard options in second-line therapy for patients with mRCC.

## Additional file

Additional file 1:
**Reasons for everolimus discontinuation and median treatment duration, TTP, and PFS by MSKCC prognostic group and mRCC histology.**

## Abbreviations

AE: Adverse event
CI: Confidence interval
HR: Hazard ratio
PFS: Progression-free survival
mRCC: Metastatic renal cell carcinoma
MSKCC: Memorial Sloan Kettering Cancer Center
mTOR: Mammalian target of rapamycin
OS: Overall survival
KPS: Karnofsky performance status
TKI: Tyrosine kinase inhibitor
TTP: Time to progression
VEGF: Vascular endothelial growth factor
