# Supplementary material for: Everolimus in metastatic renal cell carcinoma after failure of initial anti–VEGF therapy: final results of a noninterventional study
Source: BMC Cancer. 2015 Apr 18;15:303. doi: 10.1186/s12885-015-1309-7 (PMC4413536; doi:10.1186/s12885-015-1309-7)
Supplement: Additional file 1: — Reasons for everolimus discontinuation and median treatment duration, TTP, and PFS by MSKCC prognostic group and mRCC histology. [file 12885_2015_1309_MOESM1_ESM.docx]

**Supplementary Materials**

Table S1 Reasons for discontinuation of study treatment (patients who discontinued, n = 256)

| Reason^a^ | Patients, *n* (%) |
| --- | --- |
| Disease progression | 138 (54) |
| Adverse event^b^ | 54 (21) |
| Death^b^ | 36 (14) |
| Patient request | 30 (12) |
| Lost to follow-up^c^ | 17 (7) |
| Lack of efficacy | 16 (6) |
| Withdrawal of consent^c^ | 10 (4) |
| Noncompliance | 3 (1) |
| Unknown reason | 1 (<1) |

^a^Patients could have stopped therapy for multiple reasons.

^b^For four patients, adverse event and death both were specified as reasons for discontinuation.

^c^Refers to documentation; patients could have continued treatment outside of study.

When stratified by MSKCC prognostic group, median TTP and PFS tended to be longer for patients who were at intermediate or favorable MSKCC risk than for those who were at poor risk (Supplementary Table S2); however, the number of patients in the poor prognosis group was small, and no statistically significant difference was observed. When stratified by tumor histology, median TTP and PFS tended to be longer for patients with non–clear cell mRCC than for patients with clear cell mRCC (Supplementary Table S3); however, the number of patients with non–clear cell type was low, and no statistically significant difference was observed.

Abbreviations: mRCC, metastatic renal cell carcinoma; MSKCC, Memorial Sloan-Kettering Cancer Center; PFS, progression-free survival; TTP, time to progression.

Table S2. Median treatment duration, median TTP, and median PFS of everolimus by MSKCC prognostic group (efficacy population)

| MSKCC prognostic group | Treatment duration | TTP | | PFS | |
| --- | --- | --- | --- | --- | --- |
|  | Median, months (95% CI) | | | | |
| Favorable  (n = 70) | 7.6 (5–14) | 6.8 (5–20) | *p* = .48 | 6.4 (5–20) | *p* = .39 |
| Intermediate  (n = 113) | 7.9 (5–11) | 7.9 (5–10) |  | 7.4 (5–9) |  |
| Poor  (n = 19) | 3.4 (2­–14) | 4.9 (2–15) |  | 4.7 (2–15) |  |

Abbreviations: CI, confidence interval; MSKCC, Memorial Sloan-Kettering Cancer Center; PFS, progression-free survival; TTP, time to progression.

*p* value determined using log-rank test.

Table S3. Median treatment duration, median TTP, and median PFS of everolimus by mRCC histology (efficacy population)

| mRCC histology | Treatment duration | TTP | | PFS | |
| --- | --- | --- | --- | --- | --- |
|  | Median, months (95% CI) | | | | |
| Clear cell  (n = 247) | 7.4 (5–9) | 7.4 (6–9) | *p* = .84 | 7.1 (5–9) | *p* = .75 |
| Non–clear cell  (n = 17) | 3.8 (1–9) | 9.0 (5–NR) |  | 9.0 (5–NR) |  |

Abbreviations: CI, confidence interval; mRCC, metastatic renal cell carcinoma; NR, not reached; PFS, progression-free survival; TTP, time to progression.

*p* value determined using log-rank test.
